# Twelve tips for public health education using social media

**DOI:** 10.15694/mep.2021.000139.1

**Published:** 2021-05-22

**Authors:** Morgan Sehdev, Melody Huang, Nicholos Joseph, Katherine G. Nabel, Kruti Vora

**Affiliations:** 1 Harvard Medical School

**Keywords:** Social Media, Public Health Education, Twelve Tips, Instagram, Twitter, Facebook

## Abstract

This article was migrated. The article was marked as recommended.

With nearly half the world’s population using social media, platforms like Instagram, Twitter, and Facebook have become popular sources of information gathering and sharing for the general public. In medicine, social media is increasingly used to educate patients due its wide reach and interactive nature. Early studies showed that these social media-based initiatives can even promote behavioral change by increasing public knowledge and self-efficacy. Several barriers such as time and technical skills, however, prevent healthcare workers from using social media platforms to promote public health education. The following twelve tips may help reduce these barriers and create more opportunities for patients to easily access quality medical information on social media. Creating an effective public health education platform on social media involves identifying clear goals, understanding the social context of all messaging, recruiting a motivated team, creating a style guide, vetting content for accuracy, and interacting with social media followers. These tips will help build an accurate and quality social media public health education campaign.

## Introduction

Social media platforms have recently become more accessible and heavily utilized by the general population. In 2020, nearly half of the world’s population actively uses social media to connect, share, and learn ( Koetsier, 2020). Medical professionals now recognize the utility social media may have, from making professional connections to educating patients ( Ventola, 2014; Giustini *et al.,* 2018). Public health initiatives geared towards educating the general public have spread to platforms like Twitter, Facebook, and Instagram ( Kite *et al.,* 2016; Gough *et al.,* 2017; Seltzer *et al.,* 2017). Early studies have demonstrated that social media based initiatives may increase public knowledge and self-efficacy in a way that ultimately promotes behavioral change ( Xu *et al.,* 2016; An, Ji and Zhang, 2017; Daley *et al.,* 2018); however, time and necessary skills are often cited as barriers preventing healthcare workers from using social media platforms to promote public health education ( Antheunis, Tates and Nieboer, 2013). With roughly 50% of the United States’ population using social media to gather information that could affect their health ( Perrin, 2015), healthcare workers must overcome these barriers to engage with and support the public using this readily accessible resource.

The rapidly evolving nature of the COVID-19 pandemic provided a unique opportunity to disseminate public health information through social media platforms. While social media can be a source of overwhelming and inaccurate information, termed an “infodemic,” social media may also provide a far-reaching, equitable, and engaging means of educating the general public ( Schillinger, Chittamuru and Ramírez, 2020).

Hoping to provide a source of easily understandable yet data-driven information about the developing COVID-19 pandemic for the general public, a group of senior medical students created the @FutureMDvsCOVID platform. During this process, we found that there was little guidance in the literature on how to successfully design and implement a social media campaign for public health education. As a result of the lessons we learned and culled together, we formulated a series of tips for other medical trainees and providers to help develop future social media platforms with health education goals of their choosing.

## Twelve Tips

### Tip 1: Determine your goals, ideal audience, and means of communication

As a healthcare provider, you may encounter common gaps in health literacy that can be addressed using social media. Before creating your platform, it is important to explicitly define your educational goals and the audience you hope to reach, as this will drive the content you create and help provide a clear, cohesive message. For instance, when the COVID-19 pandemic began to spread in the United States, members of our team noticed common questions and misconceptions about the new virus and the situation’s gravity. Thus, we created the @FutureMDvsCOVID platform with a broad mission: to address the need for up-to-date, accessible public health education during the COVID-19 pandemic. Given the global impact of the pandemic, we sought to reach as wide an audience as possible, taking into account potential barriers such as language and social media access.

With these goals in mind, we determined our communication strategy, organizational structure, and medium of distribution. We selected Instagram and Twitter as the platforms for content distribution, as their users were within the demographics we hoped to reach. These platforms were also most conducive to the graphic design and academic nature of this work. Further extension into platforms like Facebook were pursued as bandwidth increased and more individuals were recruited to our task force.

### Tip 2: Consider how your educational goals intersect with social issues and determine your platform’s approach to these issues

Whether your platform is providing education about a chronic illness or global pandemic, it is important to recognize how social factors, including race, socioeconomic status, and education level may impact the prevalence, prevention, or presentation of the illness you aim to discuss. Health disparities persist across disease entities ( Thornton *et al.,* 2016), and public health emergencies can exacerbate pre-existing inequities. It is important to consider and address the needs of vulnerable communities in order to create effective, inclusive public health education.

While the initial mission of @FutureMDvsCOVID was focused on providing scientific information about the COVID-19 pandemic, we quickly realized that the pandemic was deeply affected by social issues, as Black, Brown, and Indigenous communities were disproportionately impacted by the virus. Other topics including the protests against mask use, the Black Lives Matter (BLM) movement, mental health, domestic violence, and insecure immigration status necessitated a public response on many social media platforms. We decided to create content that supported these social justice movements, as we believed they were deeply relevant to health equity and aligned with our goal of improving public health through education. For instance, when sharing information about proper mask use, we highlighted the increased incidence of racism that Black and Brown individuals may experience while wearing masks and urged the public to be mindful of personal biases as masks became widely used. As social media platforms exist in a dynamic social setting, do not be afraid to reevaluate your initial goals and objectives as current events unfold or as your platform develops. Just like providing information about a disease process, it is important to use scientific evidence from reliable sources (see Tip 9) when addressing social issues and to define basic terms you may use (i.e. determinants of health, racism) to provide information that is both accurate and easy to understand.

### Tip 3: Recruit a team, understand each teammate’s level of involvement, and distribute roles

In our experience, running an educational social media platform was time consuming and involved constructing posts, verifying scientific content, and responding to comments. Consider the best way to recruit a team and distribute responsibilities amongst your team members. Through email and word of mouth, @FutureMDvsCOVID recruited 85 volunteers consisting of medical students, public health students, graphic designers, data scientists, undergraduates, and PhD candidates who were excited to combat misconceptions of COVID-19. The social media platform itself also offers additional opportunities for team expansion once content is shared and distributed among your followers.

By understanding your team members’ interests, you will be able to better brainstorm how to enact your goals and divide responsibilities. Our 85 volunteers self-sorted into the following teams: content generation (writing content for posts), infographics development (designing posts), scientific review committee (vetting post content and reviewing published articles), and social media management (posting and messaging on social media). Student leads for each of these sub-committees determined their internal workflow and structure to generate content according to a common posting schedule. This reduced operational inefficiency.

### Tip 4: Design a basic “style guide”

**Figure 1:**
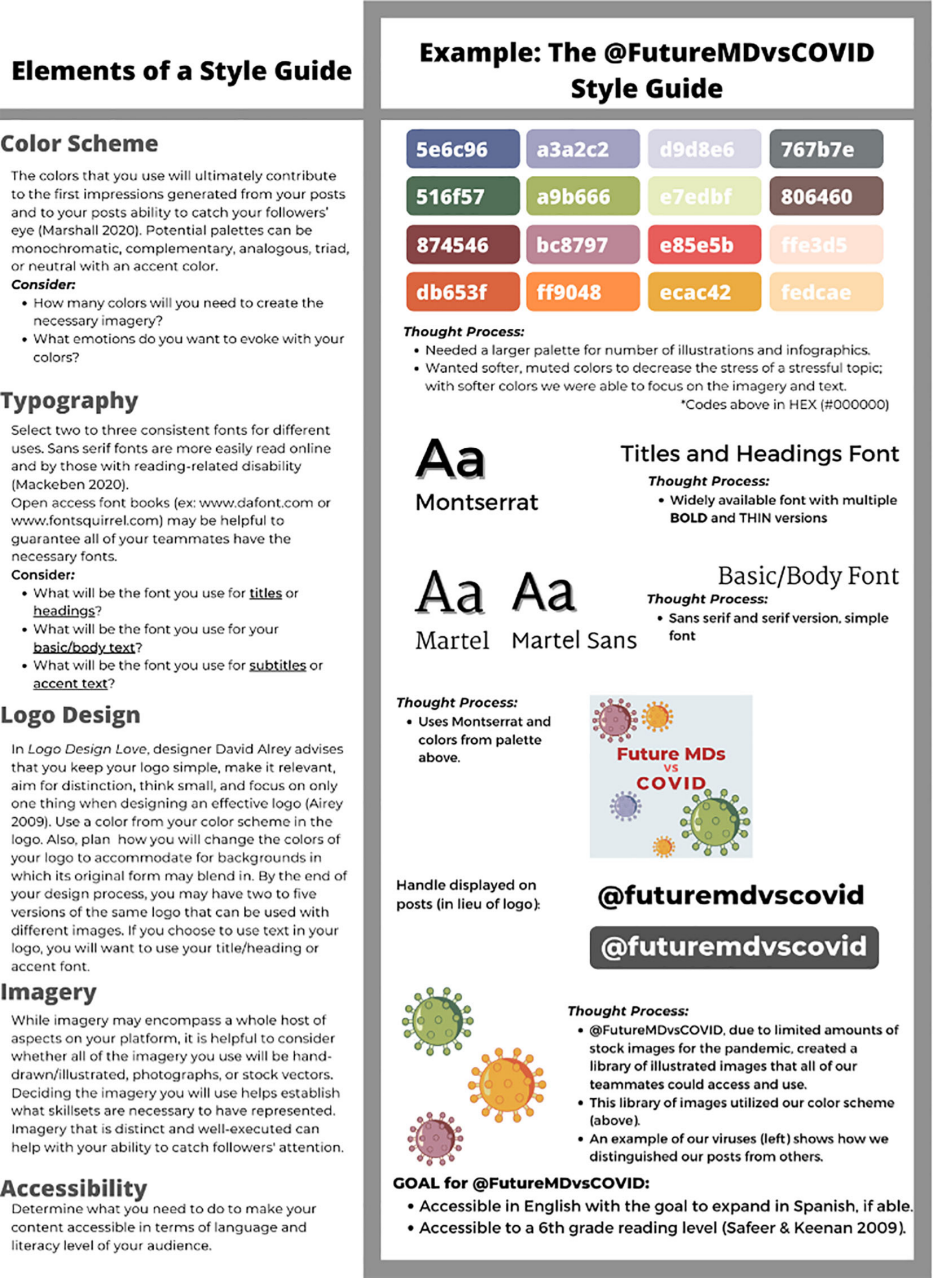
The @FutureMDvsCOVID Style Guide

Now that you’ve recruited a team, it’s time to design a post. Effective social media platforms possess an identifiable “brand” or style that creates platform recognition for the public. Many healthcare workers who would like to create visual education materials have limited design experience and inadequate resources to hire professionals. Thus, we wanted to provide the groundwork for your platform’s design. When designing a social media page for public health education, it remains important to maintain a cohesive design and branding throughout the platform, also known as a “style guide” ( Rodríguez Estrada and Davis, 2015; Arnolj, 2019), in order to aid learner engagement. To create a style guide, consider at least the following five elements: (1) color schemes, (2) typography, (3) logo design, (4) imagery, and (5) accessibility. For further description of these basic elements involved in a style guide and an example of the style guide utilized by @FutureMDvsCOVID, see Figure 1. Having a style guide allows the graphics team to work asynchronously and independently yet create a cohesive body of work. The style guide also minimizes the aesthetic decisions that must be made for each post, allowing the team to focus on the information presented as opposed to the details of design.

Five basic characteristics of a beginner style guide with examples from the @FutureMDvsCOVID style guide. The style guide can help you create a platform identity and allow you to have a coherent body of educational materials to post( Mackeben, 2000; Safeer and Keenan, 2005; Airey, 2014; Marshall, 2020) **.**

### Tip 5: Identify the tools and resources that will help create your posts

**Figure 2:**
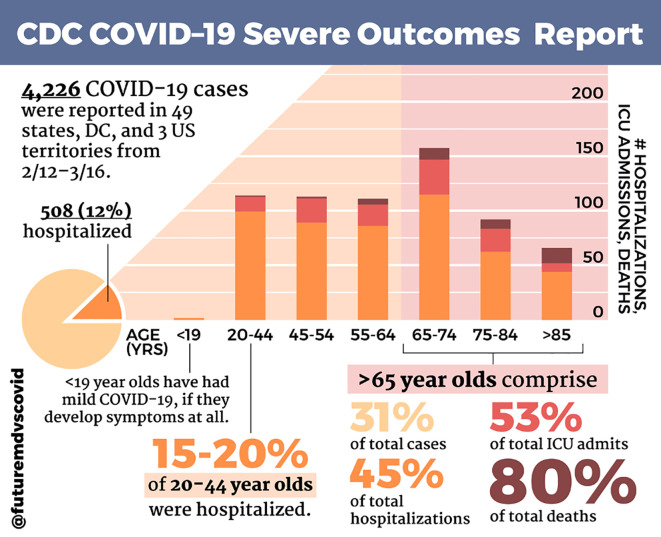
Sample @FutureMDvsCOVID Infographic

Upon creating a style guide, it is necessary to select the tools and resources that will be used to create posts or infographics. For those with limited graphic design experience, resources range from free, open access platforms or websites to full-fledged graphics programs. Our team selected programs that would accommodate differing skill sets on the team while also interfacing easily. Experienced team members were able to use Adobe programs such as Adobe Photoshop (version CC 2019) to design the imagery and format various posts. Those without access to Adobe products utilized the website Canva ( www.canva.com), as Canva allows for cloud-like sharing between teammates. Those who did not use Adobe to create images employed tablet-based applications such as Procreate (Savage Interactive, v5.0.2) and Sketchbook Pro (Autodesk Inc. v8.4.3) to draw the images that would ultimately be imported to Canva or Photoshop. For an example of a completed post designed with Canva and Adobe Photoshop, see Figure 2.

This screenshot of a @FutureMDvsCOVID infographic summarizes the CDC COVID-19 Severe Outcomes Report from February 12-March 16, 2020.Text and graphics were created using Canva (Canva, Sydney, AU) and Adobe Photoshop (Adobe Inc., San Jose, CA) according to the @FutureMDvsCOVID style guide (see Figure 1). Information from the Centers for Disease Control and Prevention, a trusted scientific source (see Tip 9), was summarized and presented in a visual, digestible manner. This infographic showed the impact of COVID-19 on different age groups within the United States.

### Tip 6: Design and utilize pre-formatted styles or templates

Once you have determined the basics of your platform’s style guide and how you will execute these designs, it is beneficial to determine what, if any, “pre-formatting” or templating can be done for the various types of posts you hope to create. By templating some of your posts, you can efficiently develop posts while allowing users to easily recognize your platform in their social media feeds ( Jof, 2020). For instance, the @FutureMDvsCOVID leadership determined that each week there would be themes for certain posts which included “Tuesday Trends” and “Fact or Fiction Friday.” Designing basic templates for these predetermined posts allowed final products to be created rapidly. Additionally, you may consider pre-formatting stock images that may be used in multiple posts. We designed several vector images of viruses, COVID-19 symptoms, and personal protective equipment. By identifying the images you can make in advance, you will enable posting on a more regular basis and promote cohesiveness across your posts.

### Tip 7: Select what topics you want to highlight in your posts using your original goals and values

Now that you have your goals, a motivated team, and a style guide for posting, what is the content you will cover? Clarifying what your target audience needs and wants to know can help identify knowledge gaps and perceived learning goals ( Boyd, 1987; Buxton, 1999; Thomas *et al.,* 2015). @FutureMDvsCOVID performed a needs assessment by asking our team members’ social media followers what questions they had about the COVID-19 pandemic. In total, we gathered 68 unique questions, which our team members then answered with supporting evidence. Our volunteers prioritized developing posts about the questions that were most frequently asked.

Timely and up-to-date content is essential. Consider whether you can continue an ongoing needs assessment of your audience as your following expands and learning gaps develop. For instance, we continued collecting comments, accepting direct messages, and hosting an open question forum on our social media platforms to identify followers’ questions as the pandemic evolved.

### Tip 8: Generate and polish content so that it is inclusive of your audience’s diversity

Once you have established the content for your social media campaign, consider how the information you are sharing is accessible and inclusive of your audience’s diversity. Most recent English and Spanish surveys of literacy levels in the United States show that 53% of adults have an intermediate level of health literacy while 36% of adults have a basic or below basic health literacy level ( Kutner *et al.,* 2006). Make your posts succinct with plenty of visuals. Consider establishing a grade-level reading standard for all your posts.

Give thought to how your posts may impact followers from different backgrounds. @FutureMDvsCOVID promoted mask use and offered low-cost DIY mask guidelines, keeping in mind some individuals may not have the means to buy a mask. We also published mask guidelines that aimed to recognize implicit biases as noted in Tip 2.

Also, recognize the nuances of different types of social media sites and the varied audiences active on each. Our Instagram audience was younger and more international. We tried to captivate this audience by initially posting memes and targeting messaging for younger people to stay home, wash their hands, wear masks, and be careful around higher-risk, older populations. We also utilized the visual structure of Instagram by showcasing our Spotlight series featuring frontline healthcare workers and COVID-19 patients. Twitter, a platform with a more academic audience, featured threads with more in-depth explanations of COVID-19 studies.

### Tip 9: Share transparent, accurate information from trusted scientific sources

As educational content is shared widely across social media, it is important to ensure the credibility and trustworthiness of all content. At the beginning of the COVID-19 pandemic, a dearth of peer-reviewed scientific articles on pressing topics led to confusion about what information could and should be shared reliably. Later, as the floodgates of pre-print articles began to open, @FutureMDvsCOVID found it important to establish ground rules for what our platform would share from various medical and scientific sources.

Our group sought to uphold the common standards of journalistic integrity when sharing scientific information on social media, striving for accuracy, transparency, and objectivity. While our group intended to have a formal faculty review process, we found this untenable given the demands on medical faculty during the pandemic and the rapid pace at which we sought to disseminate information. A team of “student vetters” was formed to verify the accuracy and reliability of scientific information in each post. Akin to the scientific review process ( Kelly, Sadeghieh and Adeli, 2014), our group used peer review from at least two to three independent people who could use scientific questioning to verify each article we shared. We collaborated with other local graduate schools to draw upon experts in our community, creating a peer-review community of MD, PhD, and MPH students, who could provide expertise to best verify each @FutureMDvsCOVID post. We agreed not to share any claim made in isolation that could not be verified by at least two independent sources. The student review team also agreed to cite peer-reviewed articles published in reliable medical and scientific journals. We avoided sharing pre-print articles given issues surrounding the lack of a formal peer-review process. If it was important to share a breaking topic that could only be cited by pre-print articles (for example, when anosmia was first reported as a COVID-19 symptom), we first determined whether the claim could be supported by two independent sources, and if so, we would disclose that the article being shared was not peer-reviewed and explain the corresponding caveats. No matter the source of the article, we felt it was always important to highlight the key strengths and limitations of each article shared. The guiding principles outlined above enabled us to more confidently share transparent, accurate information from trusted scientific sources during a moving global pandemic.

### Tip 10: Design your social media platform using descriptive and informative language and set a timeline for posting content

In order for your message to be impactful, design your platform such that it 1) clearly states your goals, 2) highlights why your platform is unique, and 3) states your qualifications for sharing such content. This will help your audience determine why they should follow you. Choose a public username for your social media accounts, also known as a “handle,” that is memorable and relevant to your mission. For instance, we chose @FutureMDvsCOVID to relate our work to the COVID-19 pandemic and mobilize the worldwide community of medical trainees. As health prevention information on social media is more likely to be shared when posted by organizations rather than individual users, consider framing your platform as an organization with legitimate qualifications to effectively share health information ( Zhang *et al.,* 2019). Our platform was granted a “Public Figure” status on Instagram shortly after we started posting.

While there is limited data to our knowledge on the ideal frequency of posts, we recommend posting at least once or twice a day to keep followers engaged when starting your page. Try to space out multiple posts during the day. Ideal posting times may depend on the location of your following. As your audience increases, Instagram has an “Insights” function that provides an idea of what times of day a page’s audience is most active. Make sure that the first few posts establish and meet the specific goals of the page. We used our first post to introduce the mission of our initiative, how it uniquely addresses the issue of misinformation during a pandemic, and why we as medical students were in a position to tackle this issue. This clear introduction helped attract followers who resonated with our message.

### Tip 11: Collaborate with institutions and other social media platforms to build an audience

Much of a platform’s impact is dependent upon the number of people it reaches. Utilize your social and institutional networks and collaborate with other platforms to broadly share your message. When doing so, emphasize the necessity of the platform’s mission and the impact you hope to create. We were able to rapidly grow our following within the first few days by encouraging all students involved with the effort to share the pages with their individual social circles, including friends and family outside the medical field. As much of our intended audience was of a younger demographic, we reached out to other popular social media pages, including those of “influencers,” to help share our message with the common goal of promoting public health and safety. We also engaged with other platforms created by health professionals and students beyond our own institution to promote ways the public could help during the pandemic, highlight frontline healthcare workers, and share additional scientific information. Using hashtags (i.e. #COVID19, #pandemic) can also allow your post to be discovered by users searching for related information.

### Tip 12: Plan for direct questioning and comments on your posts

When public health social media campaigns engage with medical experts, they build user trust in the reliability of their content, which can influence follower public health behaviors (Dixon, 2016). It is this interactive aspect of social media that makes it particularly powerful. Thus, it is important to be proactive and thoughtful when responding to comments and messages on your platform. In our experience, these included questions surrounding the pandemic, supportive remarks about our work, as well as critical commentary on our content.

Critical commentary on social media occurs frequently, particularly on platforms commonly used for academic communication, such as Twitter. This can be a powerful tool to ensure accuracy of information presented, but the quickly changing nature of information on social media and lack of formal content vetting for accuracy also increases the risk of propagating misinformation online ( Choo *et al.,* 2015). To navigate this, try to create a platform culture encouraging thoughtful and critical discussion while actively dispelling misinformation. A member of our social media team was dedicated to responding to most comments and messages while relaying critiques and more complicated questions to the leadership team for further discussion. The leadership team then worked in close conjunction with the scientific review committee to curate accurate responses and fact check posted content. If edits to our posts were needed, we would archive the original post and clarify the changes we made in order to prevent further confusion. While little data is available on the best way to correct errors on social media, adding dates and version numbers to the corrected resources may be a useful way to inform followers of the updated information ( Edwards and Roland, 2019). We deliberately set boundaries of engagement with comments that were hostile to safeguard the collaborative environment on our platform.

It is also important to acknowledge limitations to what information you can provide when responding to comments. Given that information about COVID-19 is evolving, we often cited the general limitations of the broader scientific community’s knowledge regarding the pandemic, as well as our own limitations as advanced medical students. We also acknowledged that we were unable to provide medical advice when asked on multiple occasions. With regards to any engagements with followers on social media, healthcare professionals should remain honest, as a core principle both in medicine and in guidelines for social media use ( Kind, 2015).

## Conclusion

At the outset of the COVID-19 pandemic, the American population faced uncertainty, largely driven by the lack of publicly available knowledge and evidence. As medical students equipped with an understanding of social media platforms, our group sought to leverage our expertise in dissecting medical and scientific literature to provide up-to-date, accessible public health education and community activism during the COVID-19 pandemic. We recruited a team of volunteers, assigned roles within sub-groups, and designated social media platforms for our public health education. We designed a cohesive style guide to unite our posts and assembled resources that enabled us to create professional, eye-catching social media posts collaboratively within a team of student graphic designers. We thought carefully about our audience: who they were, what content they wanted to see, and what information we could provide that would be the most useful to them. We crafted the content of our posts to have as broad a reach as possible and engaged with other social media platforms to build our audience. Knowing questions from our users would arise, we devised a plan to respond to direct questioning and comments on our post, answering questions whenever possible and setting clear boundaries for what questions our account could not answer. Ultimately, @FutureMDvsCOVID recruited 85 dedicated volunteers, attracted over 3000 followers, created over 190 posts, and partnered with several hospitals and domestic and international graduate schools. Now, @FutureMDvsCOVID is continuing to brainstorm new, innovative content as the COVID-19 pandemic evolves. We hope others will find these twelve tips useful for sharing medical information through social media, an emerging area in public health education.

## Take Home Messages

• To create an effective public health campaign through social media, identify your goals, recruit a team, assign roles, and choose your social media platform
• Design a style guide, collect design resources, and create pre-formatted templates for eye-catching posts
• Select topics to highlight and generate content inclusive of your audience’s diversity
• Share vetted, accurate information using clear, descriptive language
• Set a timeline for posting, collaborate to build an audience, and plan for comments and questions on your posts

## Notes On Contributors

**Morgan Sehdev**, BA, is a fourth year MD Candidate at Harvard Medical School (HMS), Boston, MA, USA.

**Melody Huang**, BA, is a fourth year MD Candidate at Harvard Medical School (HMS), Boston, MA, USA.

**Nicholos Joseph**, BA, is a third year MD Candidate at Harvard Medical School (HMS), Boston, MA, USA. ORCID ID: https://orcid.org/0000-0001-9365-879X

**Katherine Nabel**, BS, is a fourth year MD-PhD Candidate in the Harvard-MIT Health Sciences & Technology (HST) Program and the Harvard Graduate Program in Virology, Boston, MA, USA. ORCID ID: https://orcid.org/0000-0002-4429-7075

**Kruti Vora**, BA, is a fourth year MD Candidate at Harvard Medical School (HMS), Boston, MA, USA. ORCID ID: https://orcid.org/0000-0003-1708-1727
