# Supplementary material for: Twelve tips for public health education using social media
Source: MedEdPublish (2016). 2021 May 22;10:139. [Version 1] doi: 10.15694/mep.2021.000139.1 (PMC10939547; doi:10.15694/mep.2021.000139.1)
Supplement: Peer review history

# Reviewer report: Reviewer response for version 1

**DOI:** 10.21956/mep.18925.r27058

**Published:** 2021-10-05

**Authors:** John Sandars

**Affiliations:** 1 Edge Hill University

This review has been migrated. The reviewer awarded 4 stars out of 5 It is great to see a student initiated public health project in response to the COVID 19 pandemic - they are to be congratulated!The Tips are clear and useful to others wishing to design, develop, deliver and implement similar ventures. The design and development principles are mostly based on general principles but the focus on social media use is to be welcomed and adds to the usual principles applied to websites.

Reviewer Expertise:

NA

No decision status is available

# Reviewer report: Reviewer response for version 1

**DOI:** 10.21956/mep.18925.r27057

**Published:** 2021-06-09

**Authors:** Keith Wilson

**Affiliations:** 1 Dalhousie University

This review has been migrated. The reviewer awarded 4 stars out of 5 The present paper has been written by medical students who were interested in promoting sound COVID-19 related information using social media platforms. The paper itself summarizes the wisdom gained by these students in creating a social media presence, @FutureMDsvsCOVID, in a 12-tips style.The authors present a very organized approach that could be used by other organizations or individuals hoping to create a credible social media presence. Although targeting public health messaging, this article can be easily adapted for general development of social media messaging in other contexts. The guide presents the reader with very practical tips that are implementable by many teams. Using their own experience as a key example of the application of the tips really grounds the paper in real experience. The style guide in Figure 1 will be of particular interest to readers.The paper could be strengthened by clarifying some terminology: the word ‘platform’ is used in two contexts, namely the interactive technologies used (i.e., Twitter and Instagram) and the development of a social media presence. I would recommend using ‘presence’ in much of the text to help differentiate the meaning. Alternatively, defining early on in the paper the distinction between these two entities may help the reader.Given the widespread use of social media, it may also be helpful for the authors to provide the reader with some ideas on how to evaluate success of embarking on these types of initiatives (e.g., metrics, etc.). Additionally, where there is so much sharing of resources on these platforms, readers should be aware of copyright issues that can arise.This article should be read broadly – it is very well written and contains a very practical and organized approach to developing a social media presence. The authors are to be commended for this work, not only for the paper itself but for creating @FutureMDsvsCOVID and engaging the world on COVID issues in an evidence-informed way!

Reviewer Expertise:

NA

No decision status is available
